# A Split-Ubiquitin Based Strategy Selecting for Protein Complex-Interfering Mutations

**DOI:** 10.1534/g3.116.031369

**Published:** 2016-07-05

**Authors:** Thomas Gronemeyer, Julian Chollet, Stefan Werner, Oliver Glomb, Anne Bäuerle, Nils Johnsson

**Affiliations:** Department of Biology, Institute of Molecular Genetics and Cell Biology, Ulm University, D89081, Germany

**Keywords:** split-ubiquitin, mutagenesis, protein-protein interaction, protein structure

## Abstract

Understanding the topologies and functions of protein interaction networks requires the selective removal of single interactions. We introduce a selection strategy that enriches among a random library of alleles for mutations that impair the binding to a given partner protein. The selection makes use of a split-ubiquitin based protein interaction assay. This assay provides yeast cells that carry protein complex disturbing mutations with the advantage of being able to survive on uracil-lacking media. Applied to the exemplary interaction between the PB domains of the yeast proteins Bem1 and Cdc24, we performed two independent selections. The selections were either analyzed by Sanger sequencing of isolated clones or by next generation sequencing (NGS) of pools of clones. Both screens enriched for the same mutation in position 833 of Cdc24. Biochemical analysis confirmed that this mutation disturbs the interaction with Bem1 but not the fold of the protein. The larger dataset obtained by NGS achieved a more complete representation of the bipartite interaction interface of Cdc24.

The last few years have witnessed a dramatic increase in the amount of recorded protein interactions (Chatr-Aryamontri *et al.* 2015). A graphical display of these interactions shows that proteins are organized in highly connected networks (Han *et al.* 2004; Schwikowski *et al.* 2000). To understand the topology and logic of these networks, methods to specifically eliminate individual interactions are required (Costanzo *et al.* 2009; Sahni *et al.* 2013; Breker and Schuldiner 2014; Johnsson 2014). Finding mutations in a protein that interfere with only one or a small subset of its interaction partners often involves time-consuming approaches that depend on the specific nature of the investigated interaction (Amberg *et al.* 1995; Tian *et al.* 2014). However, the complexity of the networks requires systematic, unbiased, and large-scale compatible selection approaches to identify interaction-interfering mutations for each pair of proteins (Costanzo *et al.* 2009). Several approaches already address this need by selecting mutant libraries for interaction-defective alleles (Charloteaux *et al.* 2011; Melamed *et al.* 2015). Some of these approaches often necessitate multiple transformation and selection steps, thus greatly limiting the number of individual clones that can be screened simultaneously. In addition, the diversity of protein interactions encountered in each network clearly requires multiple and novel approaches to comprehensively dissect them.

Cdc24 is the GEF for the small Rho GTPase Cdc42 in the budding yeast *Saccharomyces cerevisiae* (Etienne-Manneville 2004). Cdc24 interacts through its C-terminal PB domain (PB_Cdc24_) with the PB domain of the scaffold protein Bem1 (PB_Bem1_) (Ito *et al.* 2001). The NMR structure of the PB_Cdc24_/PB_Bem1_ complex was solved (Ogura *et al.* 2009). The PB domain of Cdc24 uses two acidic clusters, acidic cluster 1 and 2 (Yoshinaga *et al.* 2003), that match a positively charged cluster on the PB domain of Bem1. Yoshinaga and colleagues characterized mutations in these clusters that selectively disrupt this complex (Terasawa *et al.* 2001; Yoshinaga *et al.* 2003; Ogura *et al.* 2009). Using this well described interaction as an example, we established a split-ubiquitin selection strategy to identify mutations that disrupt a given protein interaction without dramatically altering the protein’s structure.

## Materials and Methods

### Preparation and selection of the library

Mutagenesis of Cdc24_428-854_ was performed via PCR using the base analogs 2’-Deoxy-P-nucleoside-5′-Triphosphate (dPTP) and 8-Oxo-2’-deoxyguanosine-5′-Triphosphate (8oxo-dGTP) (TriLink Biotechnologies) as described elsewhere (Zaccolo *et al.* 1996). The final PCR product was cloned into a pRS313-based plasmid containing a P*_MET17_* promoter and the C_ub_-RUra3 cassette (Sikorski and Hieter 1989; Hruby *et al.* 2011), and the ligated library was electroporated into the *Escherichia coli* strain XL1-Blue. Library DNA was obtained by large-scale plasmid isolation.

High efficiency transformation of the N_ub_-Bem1 expressing yeast strain with the library DNA was performed as described elsewhere (Gietz and Woods 2002). The transformed cells were directly transferred in liquid selection medium (SD medium lacking histidine, uracil, and methionine, and containing 50 µM CuSO_4_ and 200 µg/ml geneticin). After 24 hr, aliquots of 1.5 ml of the selection mixture were pelleted and stored for plasmid isolation. A further 5 ml were pelleted, resuspended in fresh selection medium, and subjected to another round of selection.

From each round of selection, plasmid DNA was isolated and retransformed into *E. coli*. Templates for Sanger sequencing were prepared from positive clones through rolling circle amplification by an external service provider (Seqlab Laboratories). Template amplicons for NGS were PCR amplified from library plasmid DNA and plasmids isolated from a separate selection experiment. Preparation of index and adapter sequence-tagged amplicon fragments was subsequently performed with the Nextera XT kit (Illumina) according to the manufacturer’s recommendations.

Sequencing was performed with a Miseq nano v2 flow cell (Illumina) on a Miseq sequencing device (Illumina) according to the manufacturer’s instructions. Alignments and variant calling was performed using the Mapmuts software package (Bloom 2014). Enrichment scores were subsequently calculated by spreadsheet analysis and the graphical visualization of the data was performed in R Studio.

### Manual split-ubiquitin and SPR assays

JD53 cells expressing either N_ub_-Bem1 or N_ub_-ha were transformed with the plasmids carrying the respective CRU fusions. Cells were grown in selective media and serial dilutions were spotted on either nonselective media or media lacking histidine and uracil and containing various Met concentrations and 50 µM CuSO_4_. Cells were grown for 2 d at 30°.

PB_Bem1_-SNAP fusion proteins, PB_Cdc24_ and PB_Cdc24_(D833G), were expressed as 6His-tagged proteins in the *E. coli* strain BL21DE3. Purification was achieved by IMAC and optional size exclusion chromatography. All proteins were buffered in HBSEP (10 mM HEPES, 150 mM NaCl, 3 mM EDTA, 0.05% Tween 20, pH 7.4) and binding affinities were measured by SPR using a Biacore X100 system (GE Healthcare), essentially as described elsewhere (Renz *et al.* 2013). Briefly, purified PB_Bem1_-SNAP (ligand protein) was covalently labeled with BG-Biotin (New England Biolabs) by SNAP tag chemistry and captured on a CM5 SPR chip (GE Healthcare) that was previously coated with an anti-biotin antibody (US-Biologicals). For the determination of kinetic parameters, purified PB_Cdc24_ analyte protein was prepared in suitable concentrations in HBSEP buffer. Kinetic constants were calculated with the Biacore X100 Evaluation Software (Version 1.1; GE Healthcare).

### Data availability

Supplemental Material, File S1 contains a detailed protocol section. File S2 contains all Sanger sequencing data and alignments in *.clc format. A free reader can be downloaded from www.clcbio.com. NGS sequencing raw data are publically available in the European Nucleotide Archive under the following link: http://www.ebi.ac.uk/ena/data/view/PRJEB13825.

## Results and Discussion

**Figure 1:**
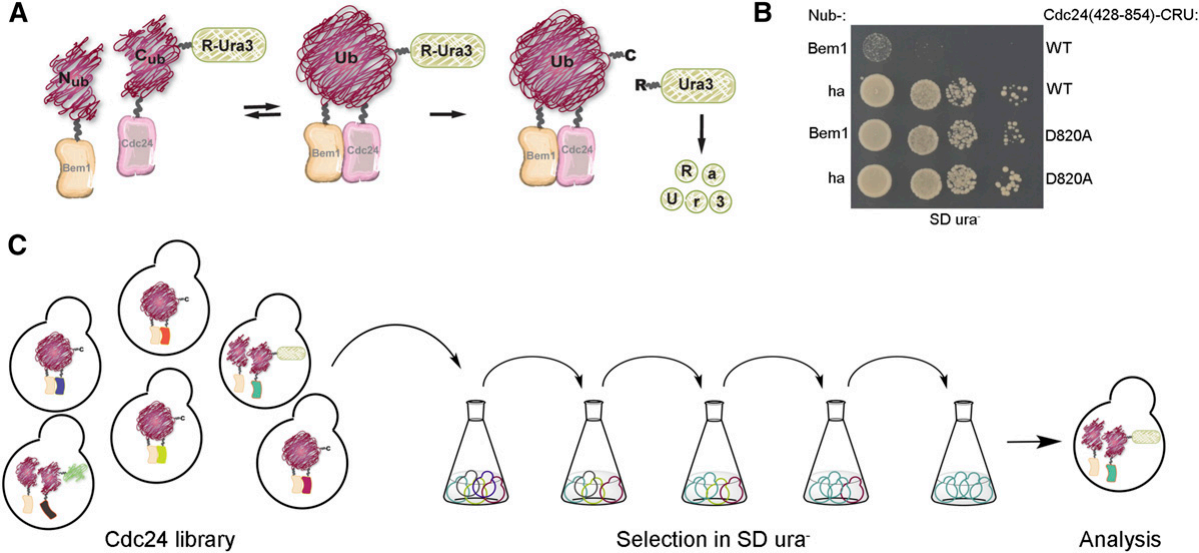
Selection strategy to enrich for interaction-interfering mutations. (A) Cartoon of the split-ubiquitin assay. Interaction between Cdc24-C_ub_-RUra3 and N_ub_-Bem1 leads to degradation of the RUra3 reporter. Cells expressing the interacting wild-type proteins as N_ub_ and C_ub_ fusions will thus stop growing on uracil-deficient medium, whereas cells expressing noninteracting mutants will continue dividing. (B) Manual split-ubiquitin assay. Cells expressing the indicated N_ub_ fusions and Cdc24_428-854_-CRU containing, at position 820, either D or A, were spotted in 10-fold serial dilutions onto SD ura^-^. Growth was recorded after 2 d at 30°. (C) Selection scheme. A library of Cdc24_428-854_ mutants fused to CRU is transformed into yeast cells expressing N_ub_-Bem1 (left) and selected in liquid SD ura^-^ (center). Clones expressing mutants of Cdc24 that do not interact with Bem1 and still display full Ura3 activity are enriched (represented by light green symbols) and analyzed. A, alanine; C_ub_, C-terminal fragment of ubiquitin; D, aspartate; N_ub_, N-terminal fragment of ubiquitin; SD ura^-^, SD medium lacking uracil; Ub, ubiquitin; WT, wild-type.

The split-ubiquitin method is based on the ability of a N-terminal (N_ub_) and a C-terminal (C_ub_) fragment of Ubiquitin (Ub) to refold into the native-like Ub upon close contact (Johnsson and Varshavsky 1994; Müller and Johnsson 2008). In our example, close contact is achieved by coupling N_ub_ and C_ub_ to Bem1 and Cdc24, respectively. The binding of the two proteins will accelerate the refolding of the coupled Ub-fragments. As a consequence, Ub-specific protease will cleave off the RUra3 reporter protein that was genetically fused to the C-terminus of C_ub_ (C_ub_-RUra3, CRU) (Figure 1A) (Wittke *et al.* 1999). Ura3 is required for uracil synthesis in yeast. After cleavage from C_ub_, the exposed destabilizing N-terminal arginine of RUra3 will lead to its rapid degradation. Yeast cells expressing Cdc24-CRU and N_ub_-Bem1 will thus stop growing on medium lacking uracil (SD ura^-^; Figure 1B). As an example for a pair of noninteracting proteins, we coexpressed Cdc24-CRU together with a N_ub_ fusion to the ha-epitope (N_ub_-ha). These cells survive on uracil-lacking media (Figure 1B). In this and all subsequent selection experiments we used the C-terminal 426 residues of Cdc24, including its C-terminal PB domain (Cdc24_428-854_) instead of the full-length protein. A D→A conversion at position 820 of Cdc24_428-854_ is known to disrupt the interaction with Bem1 (Yoshinaga *et al.* 2003). Consequently, cells coexpressing Cdc24_428-854_(D820A)-CRU together with N_ub_-Bem1 survive on SD ura^-^ (Figure 1B). Accordingly, growth in uracil-lacking medium should enrich for those clones from a pool of Cdc24 mutants that disturb the interaction with Bem1. Importantly, the selection should bias against mutations that globally interfere with the folding of Cdc24 or interrupt its reading frame, as truncated or misfolded fusion proteins should either yield no or not enough Ura3 activity for successful competition with strictly interaction-interfering mutations. Our strategy to select for interaction-interfering mutations is summarized in Figure 1C.

**Figure 2:**
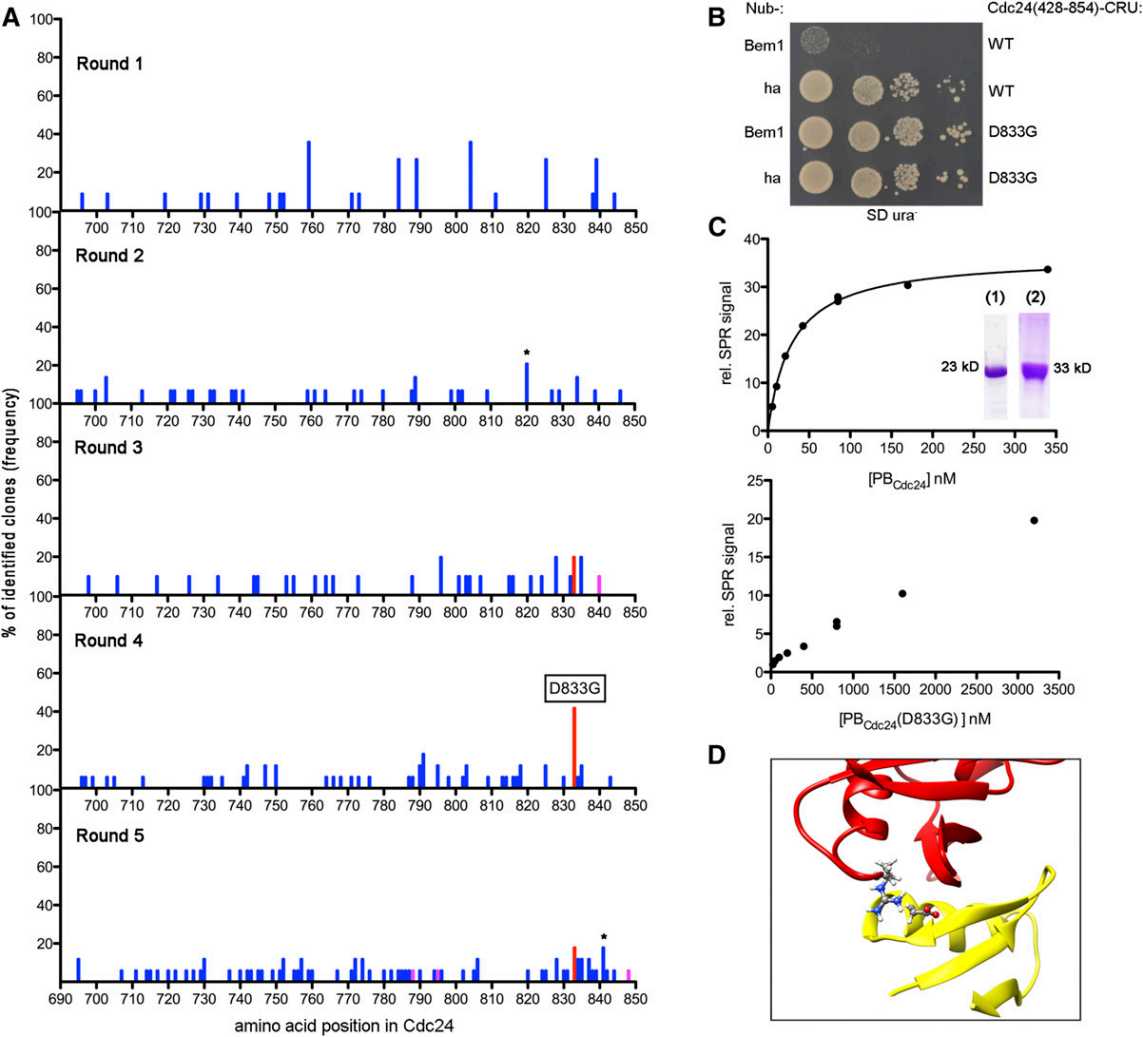
Enrichment of an interaction-interfering alteration in Cdc24. (A) Sequence analysis of each selection round. Positions of mutations in Cdc24 and their frequencies (% of identified clones) are represented as bars. The D833G mutation is shown in red. Deletion mutants are shown in magenta. Here, the position of the bar indicates the start site of the in-frame deletion. Bars labeled with an * indicate an accumulation of independent mutations and not a hotspot. (B) Manual split-ubiquitin assay of the enriched D833G mutation as in Figure 1B. (C) SPR analysis of the interaction between PB_Cdc24_ and immobilized PB_Bem1_-SNAP. Representative plots of the SPR signal *vs.* the used concentrations including the fitting curve for K_D_ determination are shown for PB_Cdc24_ (upper frame) and its D833G mutant (lower frame). The inlet shows the Coomassie-stained gel of the purified of PB_Cdc24_ (lane 1) and PB_Bem1_-SNAP (lane 2). (D) Section of the NMR structure of PB_Cdc24_ (red) and PB_Bem1_ (yellow) (PDB-ID 2KFK) highlighting the D833 and R510 residues as ball and stick presentations. NMR, nuclear magnetic resonance; SD ura^-^, SD medium lacking uracil; SPR, surface plasmon resonance; WT, wild-type.

Diversification of *CDC24_428-854_* was realized by error prone PCR in the presence of the nucleoside triphosphate analogs dPTP and 8oxo-dGTP (Zaccolo *et al.* 1996). The library insert was cloned in-frame with the CRU cassette under the control of a methionine inducible P*_MET17_*-promoter yielding a library of 2 × 10^7^ individual clones. Sequencing of randomly picked clones revealed an average of five mutations per kilobase. The library was subsequently transformed into a yeast strain expressing a genomically integrated N_ub_-Bem1 under the control of the copper inducible P*_CUP1_*-promoter, and the transformed cells were directly transferred into liquid SD ura^-^ medium. After 24 hr, a sample was taken for subsequent sequence analysis and another aliquot was diluted in fresh SD ura^-^ for the next round of selection. Five consecutive selection rounds were performed and the DNAs from at least 15 individual clones of each round were analyzed (Table 1 and File S2). A graphical display of all identified mutations is shown in Figure 2A. In round one, we spotted a number of accumulated “hotspot” mutations (each occurring in 30–40% of the sequenced clones). We refrained from classifying these mutations as interaction-interfering as none of them were recovered in the subsequent rounds.

**Table 1:** Summary of the sequence analysis of the five rounds of selection

Selection Round	Clones Sequenced	Empty Plasmid	Sequences Not Evaluated*^a^*	Sequences with Insert Evaluated	Hotspot Mutations
1	22	0	11	11	N804D (4x)
					E839G (3x)
					L828S (3x)
					F825S (3x)
					W789R (3x)
					L784W (3x)
					E759G (4x)
2	15	0	1	14	None
3	15	1	4	10	None (2x D833G)
4	20	0	2	18	D833G (7x)
5	26	6	2	18	D833G (3x)

a Some clones displayed rearrangements within the plasmid and could not be aligned to the *CDC24* sequence.

A mutation at position 833 (D833G) appeared first in selection round three and was further enriched to 40% of all evaluated clones in the subsequent round. An average of three to five mutations per clone was encountered in this round, with a few exceptions harboring a high mutation rate of up to 14 per clone. The mutations were equally distributed across the sequence.

We purified the enriched D833G exchange from the cosegregating mutations by creating a homogeneous *cdc24_428-854_(D833G)* allele through PCR. Yeast cells coexpressing Cdc24_428-854_(D833G)-CRU with N_ub_-Bem1 grew well on SD ura^-^ plates, thus confirming that this mutation is indeed responsible for the originally selected phenotype (Figure 2B). Next, we used surface plasmon resonance (SPR) of *E. coli*-expressed and purified Cdc24- and Bem1 PB domains to quantitatively measure the influence of the D833G exchange on the stability of the PB_Cdc24_/PB_Bem1_ complex. PB_Bem1_-SNAP (spanning residues 431–551) was coupled through its SNAP tag onto the surface of the SPR sensor chip. PB_Cdc24_ (spanning residues 668–854) bound to immobilized PB_Bem1_-SNAP with a K_D_ of 21 nM (± 7.8 × 10^−9^ M, n = 3) (Figure 2C). The D833G mutation [Cdc24_668-854_(D833G)] increased the K_D_ of this complex at least 160-fold above 3.2 μM (Figure 2C). This value confirms that the D833G exchange strongly impairs the tight interaction between PB_Cdc24_ and PB_Bem1_. This result is satisfyingly explained by the known structure of the PB_Cdc24_/PB_Bem1_ complex (PDB-ID 2KFK) (Ogura *et al.* 2009). Aspartate 833 is part of the second acidic cluster within the PB domain of Cdc24 that interacts with PB_Bem1_. It is located in the second α-helix. D833 of Cdc24 is in close enough proximity (3.69 Å) of R510 of Bem1 to form a stabilizing salt bridge (Figure 2D). The D→G exchange at this position will specifically eliminate this stabilizing force.

The fourth and fifth selection rounds enriched for additional missense mutations, but also for mutations that led to in-frame deletions in the PB domain or to in-frame deletions the complete *CDC24* insert. The latter two classes of mutations were not encountered in the original library or in the early selection rounds. We conclude that they most probably arose later, during the selection process in the yeast.

**Figure 3:**
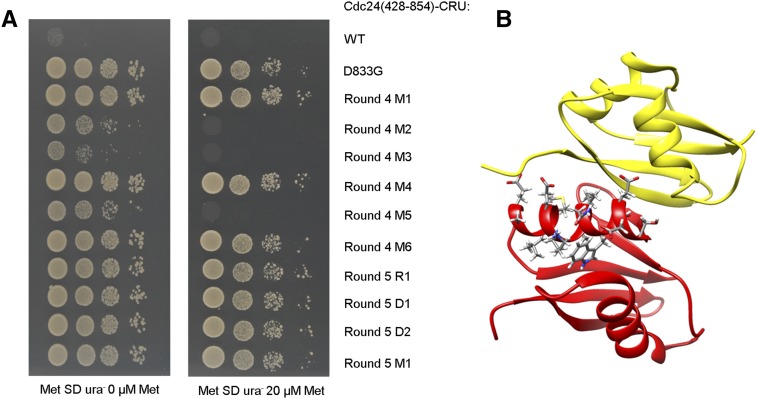
Analysis of the binding properties of mutants obtained from selection rounds 4 and 5. (A) Manual split-ubiquitin assay of the different Cdc24-C_ub_RUra3 clones (see Table 2) under conditions of high (0 µM Met, left panel) and reduced expression (20 µM Met, right panel). The medium contained 50 µM Cu to coexpress N_ub_-Bem1 in all cells. (B) Cartoon of the NMR structure of the complex between PB_Cdc24_ (red) and PB_Bem1_ (yellow) (PDB-ID 2KFK). The residues of the second helix in PB_Cdc24_, which contains D833, are highlighted as stick presentations. The first acidic cluster is located in the loop behind the helix. Cu, copper; Met, methionine; NMR, nuclear magnetic resonance; SD ura^-^, SD medium lacking uracil; WT, wild-type.

We tried to understand why the other missense mutations that were identified in selection rounds 4 and 5 were not as frequently found as the D833G mutation. Seven randomly picked clones from selection rounds 4 and 5, as well as two clones with deletions in the PB domain and one clone with a rearranged insert, were chosen for further analysis (see Table 2) and subjected to a manual split-ubiquitin assay with N_ub_-Bem1 (Figure 3). As expected, the clones bearing in-frame deletions in the PB domain or a complete rearrangement of the *CDC24* insert were able to grow on SD ura^-^. Under the conditions used for the selection, three of the missense bearing clones (M2, M3, and M5 of round 4) grew less well than the D833G mutation but still much better than the wild type. By supplying methionine into the medium we reduced the expression level of the C_ub_-fusions to make the interaction assay more stringent. Cells expressing the C_ub_-fusions M2, M3, and M5 stopped growing on SD ura^-^ medium containing 20 mM methionine (Figure 3A). We conclude that M2, M3, and M5 of round 4 still show significant binding to the PB domain of Bem1. The resulting reduction in Ura3 activity might explain the poor enrichment of these clones during the selection. The other missense mutations (M4 and M6 of round 4 and M1 of round 5) seemed to completely abolish the interaction between the two PB domains (Figure 3A). We note that all mutations that were found only once during the selection always appeared in combination with multiple other mutations in the second helix of the PB domain, or in combination with mutations in the loop preceding this helix. Both the helix and the loop, which harbors the first acidic cluster, are part of the interaction interface (Figure 3B). We infer that these mutants require at least two hits for abrogation of the interaction with Bem1. The dependency on two or more hits might explain why all other missense mutations were less enriched than D833G.

**Table 2:** Identity of the selected clones from round 4 and 5 that were analyzed by the manual split-ubiquitin assay (Figure 3A)

Clone	Mutations	Ura Sensitivity
WT	None	Yes
D833G	D833G	No
Round 4 M1	N752S, F791S, M796V, I816V, F825C*^a^*, N835D*^b^*, K838R*^b^*	No
Round 4 M2	V788I, E839G*^b^*, K847R	Yes
Round 4 M3	T701A, N790H, S803P, N809S	Yes
Round 4 M4	S830R*^a^*, W834R*^b^*	No
Round 4 M5	P705S, D730N, S744G, Y768C, F791T, K847T	Yes
Round 4 M6	R735W, K747R, I795R, S803P, Y818D*^a^*, F825S*^a^*, K838N*^b^*	No
Round 5 R1	Complete rearrangement; no insert	No
Round 5 D1	N752K, in-frame deletion from K787 on	No
Round 5 D2	N752S, N755T, I780, in-frame deletion from L794 on	No
Round 5 M1	F742S, E751G, S756P, F791L, W834R*^b^*, V836A*^b^*, M840T*^b^*, L841W*^b^*	No

Ura, uracil; WT, wild-type.

a Mutations that lie in other structural elements contributing to the interface of the PB domain of Cdc24.

b Mutations that lie within the second helix of the Cdc24 PB domain.

**Figure 4:**
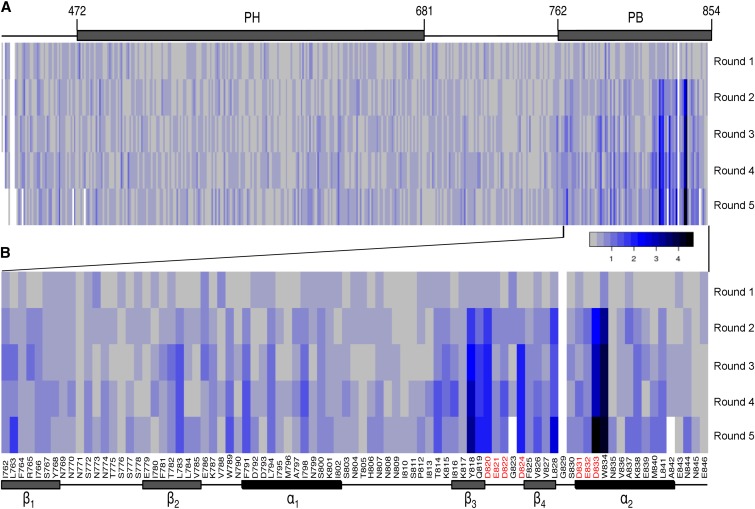
Heat map diagram of the enrichment values of each selection round obtained by NGS. (A) Entire library insert (Cdc24_428-854_). (B) Blow up of the PB domain. The white fields represent data deficient positions. Amino acid positions of the acidic clusters in the PB domain are indicated by red letters. NGS, next generation sequencing; PB, Phox and Bem1; PH, Pleckstrin homology.

The first acidic cluster of PB_Cdc24_ comprises three aspartate residues (D820, D822, and D824) located in a loop behind the second helix (Terasawa *et al.* 2001; Yoshinaga *et al.* 2003). Although the split-ubiquitin assay clearly detects the influence of the D820A exchange on the interaction between N_ub_-Bem1 and Cdc24_428-854_-CRU (Figure 1), our selection did not reveal this or any other mutation in the first acidic cluster. We reasoned that the limited amount of analyzed clones might not accurately reflect the whole spectrum of enriched mutations. Thus, we repeated the selection under identical conditions but turned to NGS for the analysis of large pools of clones (Fowler and Fields 2014). As the analysis skips the isolation of the mutation-bearing plasmids, we did not test for the plasmid dependency of the enriched phenotype (Figure 3). As a consequence, a spontaneous genomic mutation in either *N_ub_-BEM1* or any other loci that restores growth on SD ura^-^ might remain undetected and spread through the population. Thus, we mated eight randomly picked clones of each selection round against a yeast strain expressing N_ub_-Bem1. The growth on SD ura^-^ of the tested diploids excluded the significant occurrence of recessive genomic mutations in the yeast cells, and confirmed that the selection enriched primarily for mutations in the Cdc24_428-854_-CRU-containing plasmid (Figure S1). We then prepared PCR amplicons for NGS from each round of selection in such a way that deletion mutants were not amplified but removed from the analysis. Using this approach, approximately 200,000 sequence reads per selection round (before filtering) were obtained, which resulted in a read depth of a minimum of 1000 up to a maximum of 20,000 after data filtering and mapping. Figure 4 shows the heat map diagram of the enrichment values for five selection rounds. Enrichment values were obtained by the log_2_ transformation of the enrichment scores of each mutated site and subsequent combination of these scores for each selection round. The calculation is outlined in detail in the supplemental Materials and Methods section in File S1.

Clear enrichment of mutations that all cluster in the second half of the PB domain of Cdc24 has already been observed in the second round of selection (Figure 4A). A blow-up of the PB domain identifies these residues as Y818 (1.87), D820 (1.64), L828 (1.61), D833 (2.45), and W834 (3.35), with their enrichment values given in parentheses (Figure 4B). Up to selection round 5, this spectrum of mutations remains nearly unchanged with the exception of two additional sites emerging at positions 819 and 824. The enrichments for these sites after selection round 5 are: Y818 (3.14), Q819 (1.88), D820 (1.88), D824 (1.88), L828 (2.74), D833 (4.66), and W834 (4.08). The positions of these mutations nicely trace the bipartite character of the PB_Cdc24_ interaction interface. The aspartates at positions 820 and 824 are part of the first acidic cluster, and D833 falls into the second acidic cluster (Yoshinaga *et al.* 2003). These residues, together with Y818, are in direct contact with residues on the complementary interface of Bem1. The mutations at positions 819, 828, and 834 probably disturb the structure of the binding interface.

We conclude that the herein introduced methodology selects for interaction-interfering mutations. The method is not limited to yeast proteins (Dirnberger *et al.* 2008; Kundu *et al.* 2013). As a genetic selection it is unbiased, can be easily scaled up, and can be applied to a wide class of pairs of proteins including membrane proteins, transcription factors, or proteins residing on the surface of organelles (Wittke *et al.* 1999; Eckert and Johnsson 2003; Bashline and Gu 2015). Screens for interaction-interfering mutations already described include a similar split protein sensor approach based on the yeast cytosine deaminase (Dreze *et al.* 2009; Ear and Michnick 2009; Charloteaux *et al.* 2011; Melamed *et al.* 2015). We would expect that each approach biases against different sets of interactions and, thus, contributes important complementary information on the interaction network. The wider spectrum of detected mutations provides proof that the NGS approach is superior to single clone sequencing. This advantage has to be traded against the inability of NGS to recognize pairs of mutations that exert their effect only in combination.

## Supplementary Material

Supplemental Material
