# Supplementary material for: A Split-Ubiquitin Based Strategy Selecting for Protein Complex-Interfering Mutations
Source: G3 (Bethesda). 2016 Jul 5;6(9):2809–15. doi: 10.1534/g3.116.031369 (PMC5015938; doi:10.1534/g3.116.031369)
Supplement: Supplemental Material [file supp_6_9_2809__index.html]

A Split-Ubiquitin Based Strategy Selecting for Protein Complex-Interfering Mutations — Supplemental Material 

# A Split-Ubiquitin Based Strategy Selecting for Protein Complex-Interfering Mutations

## Supplemental Material for Gronemeyer *et al.*, 2016

**Files in this Data Supplement:**

- File S1 - Supplemental materials and methods. (.pdf, 298 KB)
- Figure S1 - Eight randomly picked clones from selection round five were mated either with a JD47 strain expressing Nub-Bem1 or with JD47 wild type yeast. (.tif, 1 MB)
- File S2 - Sequence alignments of the Sanger sequencing in \*.clc format (zip file). (.zip, 5 MB)
