# Supplementary material for: A Split-Ubiquitin Based Strategy Selecting for Protein Complex-Interfering Mutations
Source: G3 (Bethesda). 2016 Jul 5;6(9):2809–15. doi: 10.1534/g3.116.031369 (PMC5015938; doi:10.1534/g3.116.031369)
Supplement: Supplemental Material [file supp_g3.116.031369_FileS1.pdf]

Supplemental Materials and Methods

Plasmids and strains

A pRS313-based plasmid containing a P_{MET17}-promoter promoter and the C_{ub}-R-Ura3 cassette (pMet-CRU-313) (Sikorski and Hieter 1989, Hruby *et al.* 2011) was used for all Split-Ubiquitin based experiments. $Cdc24_{428-854}$ was PCR-amplified from genomic DNA prepared from yeast strain JD47 (*MATa his3*-Δ200 *leu2-3,112 lys2-801 trp1*Δ63 *ura3-52*) and cloned into pMet-CRU-313 using the *Eag1* and *Sal1* restriction sites. The mutations D820A and D833G were introduced by SOE-PCR. The PB domain of Bem1 (A431-I551) was PCR-amplified from JD47 genomic DNA and cloned in frame of a SNAP tag into a pET15b derived expression plasmid (Iffland *et al.* 2000, Gronemeyer *et al.* 2006). The PB domain containing the linker sequence between the PH and PB domain of Cdc24 (H668-Y854) was amplified from JD47 genomic DNA and cloned into a pET15b derived expression plasmid (Iffland *et al.* 2000). The D833G mutation was introduced by SOE-PCR. Protein expression was carried out in the *E. coli* strain BL21(DE3). Selection of the library was performed in the haploid yeast strain JD53 (genotype *MATalpha his3-Δ200 leu2-3,112 lys2-801 trp1*Δ63 *ura3-52*) containing the genomically integrated N_{ub}-Bem1 (*P_{BEM1}::kanMX6 $P_{CUP1}N_{ub}$-HA*).

Preparation of the library

Mutagenesis of $Cdc24_{428-854}$ was performed through PCR using the base analogues 2'-Deoxy-P-nucleoside-5'-Triphosphate (dPTP) and 8-Oxo-2'-deoxyguanosine-5'-Triphosphate (TriLink Biotechnologies) as described elsewhere (Zaccolo *et al.* 1996). The base analogs dPTP and 8oxo-dGTP stimulate the transitions A→G and T→C four to five times more frequently than all other exchanges (Zaccolo *et al.* 1996). This feature is reflected by the identity of our enriched D833G mutation. The required A→G transition

at position two of this codon is more probable than all other non-silent mutations.

To determine the concentration of base analogues needed for the intended mutagenesis rate of 3-5 amino acid mutations per kilobase, different dilutions of the base analogue mixture were prepared: While the ratio of dPTP to 8-oxodGTP remained constant, a mixture of 10 mM dPTP and 40 mM 8-oxo-dGTP (base-mix) was diluted with ddH_2O and subsequently used for error prone PCR. A 1:3 dilution of the base-mix and 3 cycles resulted in an acceptable mutational rate of about 3.6 amino acid substitutions per 1000 bp (data not shown).

Biotinylated forward (cctcccggccgATGTCGAGTGACGATAATAATACGAA) and reverse primers (gtatcgtcgacCCATACAGACGAATGTTCAAGAATTTC) were used for the error prone PCR. *Eag1* and *Sal1* restriction sites are underlined. For the preparation of library DNA, the PCR product was captured on streptavidin coated magnetic beads (New England Biolabs) to remove the template DNA. After washing, the beads were resuspended in 40 µl of PCR-buffer (20 mM Tris-HCl pH 8.8, 2 mM $MgCl_2$, 10 mM KCl, 10 mM $(NH_4)_2SO_4$, 0.1 % (v/v) Triton X100, 0.1 mg/ml BSA). From this suspension, 5 µl was used as template for subsequent PCR-amplification in which the same (but non-biotinylated) primers and unmodified dNTPs were used. To lower the mutational bias in the library, four independent error-prone PCRs were performed and subsequently combined for large-scale diversification. The PCR product was digested with *Eag1* and *Sal1*, purified, and ligated into the pMet−CRU-313 plasmid. Ligation was performed for 18 h at 16°C using 15.6 µg plasmid, 6.25 µg insert and 150 U T4 DNA-ligase (Thermo Fischer Scientific). After heat inactivation of the Ligase at 65°C for 15 min, the DNA was precipitated with ethanol, resuspended in ddH_2O and used for electroporation into competent XL1blue *E. coli*. The transformation of the complete library resulted in a total

of 90 large (150 mm) dishes. Each dish was flooded with 5 ml 2YT medium; the cells were scraped off the plates, adjusted to 43% glycerol and stored in aliquots at -80°C. We sequenced 12 randomly picked clones with a primer allowing us to cover the first 1000 base pairs and further 18 randomly picked clones with a primer allowing to cover the last 800 base pairs. Alignment of these sequences with the Cdc24 ORF sequence revealed an average of 5 mutations per kilobase - slightly more than estimated in the test experiments (File S2). Library DNA for transformation in yeast was prepared by diluting one aliquot of the library stock in 150 ml 2YT medium and incubating at 37°C until an OD_{600} of 1.5. Plasmid DNA was prepared form this culture by using a Plasmid Maxi Kit (Thermo Fisher Scientific).

Library transformation and selection in yeast

High efficiency transformation of the N_{ub}-Bem1 expressing yeast strain (Hruby *et al.* 2011) was performed as described elsewhere (Gietz and Woods 2002). The transformed cells were directly transferred in liquid selection medium (SD medium lacking histidine, uracil, methionine and containing 50 µM $CuSO_4$ and 200 µg/ml geneticin). After 24h, aliquots of 1.5 ml of the selection mixture were pelleted and stored for plasmid isolation. Further 5 ml were pelleted, resuspended in fresh selection medium and subjected to another round of selection.

Plasmid isolation and analysis by Sanger sequencing

Plasmid isolation from the pelleted yeast was performed with the Charge Switch Plasmid Yeast Miniprep Kit (Life Technologies) according to the manufacturer's instructions. The eluted DNA was precipitated with ethanol and subsequently electroporated into competent XL1blue *E.coli*. Positive clones were identified by colony PCR and subsequently grown in LB medium containing 100 µg/ml Ampicillin. Templates for

Sanger sequencing were prepared from these clones through rolling circle amplification by an external service provider (Seqlab Laboratories). Sequence alignments and analysis were performed with the CLC Main Workbench version 7.6.2 (CLC Bio/Qiagen) (File S2).

Plasmid isolation and analysis by Next Generation Sequencing (NGS)

Template amplicons for NGS were PCR amplified from plasmids isolated by Phenol/Chloroform extraction from a separate selection experiment that was performed under identical conditions as described above. Application of the Charge Switch Kit resulted in failure of the subsequent PCR reaction for unknown reasons. A forward primer aligning approx. 50 bp before the ORF in the pMet-CRU-313 plasmid (ctagaggatccTACATAGATACAATTCTATTACC) (a *BamH1* restriction site is underlined) and a biotinylated reverse primer aligning with the terminal codons of *CDC24* (cggtacggaattcggttgctgagcgcaggcctgcagcggccgtcgagtcgacaagcttgATACAGACGAATGT TCAAGAATTTC) were used. This design prevented the amplification of deletion mutants. The biotinylated reverse primer contained an additional 59 bp extension (minor letters) including an *EcoR1* restriction site (underlined) to generate the 50 bp extension that is necessary for NGS template preparation with the Nextera system (see below). PCR was performed for 3 cycles and further 20 cycles using annealing temperatures of 52°C and 59°C, respectively. Re-cloning in a pEGX2T plasmid via the *BamH1* and *EcoR1* sites and subsequent sequencing of a number of clones revealed that the library complexity was not altered by the PCR amplicon preparation (data not shown). The purified PCR product was captured on streptavidin coated magnetic beads to remove plasmid template DNA and eluted from the beads by restriction with *EcoR1* after washing in CutSmart buffer (New England Biolabs). The product was purified by a PCR Purification

Minelute Kit (Thermo Fisher Scientific) and quantified using the QuantiFluor dsDNA System (Promega). Preparation of index- and adapter sequence tagged amplicon fragments was subsequently performed with the Nextera XT Library Preparation Kit (Illumina) according to the manufacturer's recommendations. Size distribution of the NGS-ready amplicon fragments was monitored with a QIAxcel device (Qiagen).

Sequencing was performed with a Miseq nano v2 flow cell (Illumina) on a Miseq sequencing device (Illumina) according to the manufacturer's instructions.

Sequencing was performed as paired read runs of the input library and of each selection round. Each read of the paired end sequencing run generated two files that were retrieved from the MiSeq system.

Quality filtration of the raw reads and subsequent mapping to the reference amplicon (corresponding to the wildtype $Cdc24_{428-854}$ sequence) was performed with the mapmuts.makealignments algorithm of the Mapmuts software package (Bloom 2014). Only read pairs with an overlap of at least 25 bases and an average Phred-score quality of 28 or higher were included in the further analysis. Subsequently, amino acid identities at each position were counted using the mapmuts.parsecounts script (Bloom 2014). This script determines also the coverage at each position. Subsequent data processing was performed by spreadsheet calculation.

The first and the last positions of each sequence read pair showed a much higher mismatch rate (up to 10-fold higher, data not shown) than the rest of the read. As a consequence we removed the distal 9 bases for all sequence runs.

Very low counts in the input library might result in artificially high enrichment scores and thus only variants with amino acid counts higher than 4 were considered for further processing. Otherwise, counts were set to zero.

In average, values for three mutated amino acids were obtained at each site of the input library. Then the frequency F of each amino acid identity *i* at each position *p* was calculated by dividing the counts of the respective amino acid by the coverage at that position.

The enrichment score ES of an particular amino acid was calculated as follows:

$$ES_i = \frac{F_{i(selection)}}{F_{i(library)}}$$

Because the enrichment score of a non-mutated (wildtype) amino acid is defined as 1 (Melamed *et al.* 2013), enrichment scores were normalized to the corresponding wildtype ES at a particular position.

For better visualization of the data of multiple selection rounds and to summarize the mutational sensitivity of the different positions, we calculated enrichment values (EV) as follows: First, the normalized scores were $\log_2$ transformed. Second, the data of all normalized, transformed enrichment scores at a certain site in one selection round were combined by the following calculation: The absolute values of the $\log_2$ transformed enrichment scores were summed up and divided by the number of summands at the particular position according to the following equation:

$$EV_p = \frac{\sum_i \left| log_2 ES_{i,p} \right|}{\sum i_p}$$

The enrichment values of the different selection rounds were visualized as a heatmap diagram that was created in Rstudio (gplots package).

Manual Split-Ubiquitin assay

JD53 cells expressing either N_{ub}-Bem1 or N_{ub}-ha were transformed with the plasmids carrying the respective CRU fusions. Cells were grown in selective media to an OD_{600} of 1. Of this culture and further 10-fold serial dilutions 4.5 µl were spotted on media lacking methionine, histidine and uracil and containing 50 µM $CuSO_4$. Cells were grown for two days at 30°C. The same dilutions were also spotted on media containing uracil to control for the equal growth of the strains under non-selective conditions (Hruby *et al.* 2011, Dünkler *et al.* 2012).

Purification of recombinant proteins

Protein expression of 6His-tagged PB_{Bem1}-SNAP was carried out in SB medium at 18°C for 5 h. The cells were lysed by treatment with lysozyme and sonication. Purification was done by IMAC using an ÄKTA Purifier chromatography system (GE Healthcare). The protein was eluted from a 5ml HisTrap HP column (GE Healthcare) in a linear imidazole gradient. Fractions containing the purified protein were pooled, buffered in HBSEP (10 mM HEPES, 150 mM NaCl, 3 mM EDTA, 0.05% Tween 20, pH 7.4) containing 30% Glycerol using a PD10 desalting column (GE Healthcare) and stored until use at -20°C. Cells expressing 6His-tagged PB_{Cdc24} or PB_{Cdc24}(D833G) were cultivated in LB medium at 37°C. Cell lysis and protein purification with IMAC was performed as described above. IMAC fractions containing the respective protein were pooled and subjected to size exclusion chromatography in HBSEP buffer using a Superdex 200 16/60 column (GE Healthcare). Fractions containing the purified protein were pooled, concentrated and used directly for SPR measurements.

Determination of binding affinities

Binding affinities were measured by SPR using a Biacore X100 system (GE Healthcare)

essentially as described elsewhere (Renz *et al.* 2013). Briefly, the surface of a CM5 Chip (GE Healthcare) was coated with an anti-Biotin-antibody (US Biologicals) using NHS ester chemistry in 10 mM sodium acetate pH 5.0 with HBSEP as running buffer. Purified PB_{Bem1}-SNAP (ligand protein) was covalently labeled with BG-Biotin (New England Biolabs) by SNAP tag chemistry in HBSEP buffer and excess substrate was removed using a NAP5 desalting column (GE Healthcare). HBSEP was used as running buffer for all subsequent experiments. Capture levels of biotinylated PB_{Bem1}-SNAP on the chip were in the range of 60-100 RU. For the determination of kinetic parameters, purified PB_{Cdc24} analyte protein was prepared in suitable concentrations in HBSEP buffer. The contact time with the ligand was set between 60 and 180 s followed by a 300 s dissociation period for each cycle. Analyte titrations were performed simultaneously on the reference cell (without ligand molecule) and the detection cell. Background correction (i.e. subtraction of the reference cell signal) was performed by default. Regeneration of the sensor chip between each analyte cycles was performed with a 15 s injection pulse of 12 mM NaOH. Kinetic constants were calculated with the Biacore X100 Evaluation Software (Version 1.1; GE Healthcare).

Supplemental References

Bloom,J.D. 2014 An experimentally determined evolutionary model dramatically improves phylogenetic fit. Mol Biol Evol. 31: 1956-1978.

Dünkler,A., Müller,J. and Johnsson,N. 2012 Detecting protein-protein interactions with the Split-Ubiquitin sensor. Methods Mol Biol. 786: 115-130.

Gietz,R.D. and Woods,R.A. 2002 Transformation of yeast by lithium acetate/single-stranded carrier DNA/polyethylene glycol method. Methods Enzymol. 350: 87-96.

Gronemeyer,T., Chidley,C., Juillerat,A., Heinis,C. and Johnsson,K. 2006 Directed evolution of O6-alkylguanine-DNA alkyltransferase for applications in protein labeling. Protein Eng Des Sel. 19: 309-16.

Hruby,A., Zapatka,M., Heucke,S., Rieger,L., Wu,Y.*, et al*. 2011 A constraint network of interactions: protein-protein interaction analysis of the yeast type II phosphatase Ptc1p and its adaptor protein Nbp2p. J Cell Sci. 124: 35-46.

Iffland,A., Tafelmeyer,P., Saudan,C. and Johnsson,K. 2000 Directed molecular evolution of cytochrome c peroxidase. Biochemistry. 39: 10790-8.

Melamed,D., Young,D.L., Gamble,C.E., Miller,C.R. and Fields,S. 2013 Deep mutational scanning of an RRM domain of the Saccharomyces cerevisiae poly(A)-binding protein. RNA. 19: 1537-1551.

Renz,C., Johnsson,N. and Gronemeyer,T. 2013 An efficient protocol for the purification and labeling of entire yeast septin rods from E.coli for quantitative in vitro experimentation. BMC Biotechnol. 13: 60-6750-13-60.

Sikorski,R.S. and Hieter,P. 1989 A system of shuttle vectors and yeast host strains designed for efficient manipulation of DNA in Saccharomyces cerevisiae. Genetics. 122: 19-27.

Zaccolo,M., Williams,D.M., Brown,D.M. and Gherardi,E. 1996 An approach to random mutagenesis of DNA using mixtures of triphosphate derivatives of nucleoside analogues. J Mol Biol. 255: 589-603.
