# Supplementary figures and images for: A Split-Ubiquitin Based Strategy Selecting for Protein Complex-Interfering Mutations
Source: G3 (Bethesda). 2016 Jul 5;6(9):2809–15. doi: 10.1534/g3.116.031369 (PMC5015938; doi:10.1534/g3.116.031369)

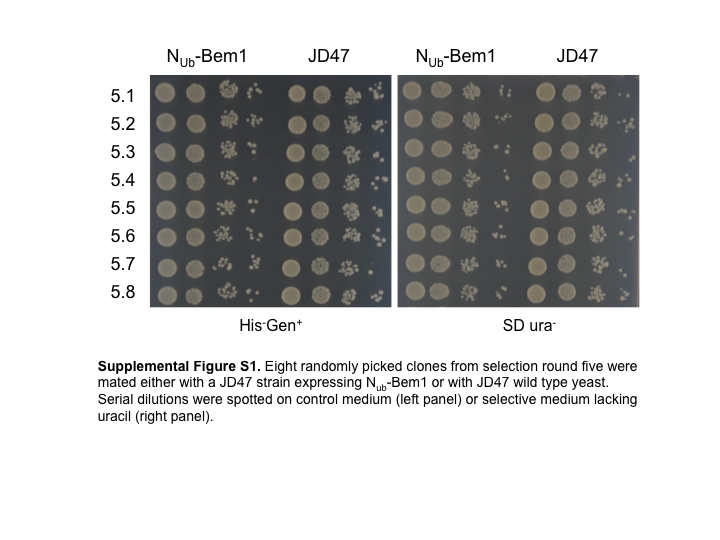

Supplement: Supplemental Material [file supp_g3.116.031369_FigureS1.tif]
